# Supplementary material for: Circuit explained: How does a transformer perform compositional generalization
Source: PLoS One. 2026 Feb 4;21(2):e0340088. doi: 10.1371/journal.pone.0340088 (PMC12871980; doi:10.1371/journal.pone.0340088)
Supplement: Peer review history

# Peer review: Decision Letter 0

**DOI:** 10.1371/journal.pone.0340088.r001

**Authors:** Constantine Dovrolis

26 Oct 2025

PONE-D-25-47812Circuit Explained: How Does a Transformer Perform Compositional GeneralizationPLOS ONE

Dear Dr. Jazayeri,

Thank you for submitting your manuscript to PLOS ONE. After careful consideration, we feel that it has merit but does not fully meet PLOS ONE’s publication criteria as it currently stands. Therefore, we invite you to submit a revised version of the manuscript that addresses the points raised during the review process.

Please submit your revised manuscript by Dec 10 2025 11:59PM. If you will need more time than this to complete your revisions, please reply to this message or contact the journal office at plosone@plos.org. When you're ready to submit your revision, log on to https://www.editorialmanager.com/pone/ and select the 'Submissions Needing Revision' folder to locate your manuscript file.

Please include the following items when submitting your revised manuscript:

- A rebuttal letter that responds to each point raised by the academic editor and reviewer(s). You should upload this letter as a separate file labeled 'Response to Reviewers'.
- A marked-up copy of your manuscript that highlights changes made to the original version. You should upload this as a separate file labeled 'Revised Manuscript with Track Changes'.
- An unmarked version of your revised paper without tracked changes. You should upload this as a separate file labeled 'Manuscript'.

If you would like to make changes to your financial disclosure, please include your updated statement in your cover letter. Guidelines for resubmitting your figure files are available below the reviewer comments at the end of this letter.

If applicable, we recommend that you deposit your laboratory protocols in protocols.io to enhance the reproducibility of your results. Protocols.io assigns your protocol its own identifier (DOI) so that it can be cited independently in the future. For instructions see: https://journals.plos.org/plosone/s/submission-guidelines#loc-laboratory-protocols. Additionally, PLOS ONE offers an option for publishing peer-reviewed Lab Protocol articles, which describe protocols hosted on protocols.io. Read more information on sharing protocols at https://plos.org/protocols?utm_medium=editorial-email&utm_source=authorletters&utm_campaign=protocols.

We look forward to receiving your revised manuscript.

Kind regards,

Constantine Dovrolis

Academic Editor

PLOS ONE

Journal Requirements:

When submitting your revision, we need you to address these additional requirements.

1.Please ensure that your manuscript meets PLOS ONE's style requirements, including those for file naming. The PLOS ONE style templates can be found at

https://journals.plos.org/plosone/s/file?id=wjVg/PLOSOne_formatting_sample_main_body.pdf and

https://journals.plos.org/plosone/s/file?id=ba62/PLOSOne_formatting_sample_title_authors_affiliations.pdf

2. We note that the grant information you provided in the ‘Funding Information’ and ‘Financial Disclosure’ sections do not match.

When you resubmit, please ensure that you provide the correct grant numbers for the awards you received for your study in the ‘Funding Information’ section.

3. Thank you for stating the following financial disclosure:

“C.T. is supported by Friends of the McGovern Institute Student Fellowship.

M.J. is supported by the Simons Foundation, HHMI and the McGovern Institute.”

Please state what role the funders took in the study.  If the funders had no role, please state: "The funders had no role in study design, data collection and analysis, decision to publish, or preparation of the manuscript."

If this statement is not correct you must amend it as needed.

Please include this amended Role of Funder statement in your cover letter; we will change the online submission form on your behalf.

4. Thank you for uploading your study's underlying data set. Unfortunately, the repository you have noted in your Data Availability statement does not qualify as an acceptable data repository according to PLOS's standards.

At this time, please upload the minimal data set necessary to replicate your study's findings to a stable, public repository (such as figshare or Dryad) and provide us with the relevant URLs, DOIs, or accession numbers that may be used to access these data. For a list of recommended repositories and additional information on PLOS standards for data deposition, please see https://journals.plos.org/plosone/s/recommended-repositories.

5. If the reviewer comments include a recommendation to cite specific previously published works, please review and evaluate these publications to determine whether they are relevant and should be cited. There is no requirement to cite these works unless the editor has indicated otherwise. 

6. Please review your reference list to ensure that it is complete and correct. If you have cited papers that have been retracted, please include the rationale for doing so in the manuscript text, or remove these references and replace them with relevant current references. Any changes to the reference list should be mentioned in the rebuttal letter that accompanies your revised manuscript. If you need to cite a retracted article, indicate the article’s retracted status in the References list and also include a citation and full reference for the retraction notice.

Additional Editor Comments:

Your manuscript “Circuit Explained: How Does a Transformer Perform Compositional Generalization” has been evaluated by two reviewers. Both reviewers found the study methodologically sound, well executed, and clearly written. They particularly commended the rigorous use of causal ablation and path-patching to identify a minimal attention circuit that explains compositional behavior in a compact transformer.

However, both reviewers raised several points that should be addressed before the paper can be accepted.

1. Scope and claim calibration: Please align the statements in the Abstract and Introduction with the limitations already acknowledged in Section “Limitations and Future Work.” The paper should emphasize that the circuit was identified in a small, synthetic task and in a limited number of model instances.

2. Dataset clarification: Clarify whether the 12,000 total episodes represent unique function–argument combinations and whether diversity in training episodes affects the emergence of the circuit. A brief comment or supplementary analysis would suffice.

3. Generality of findings: Expand the Discussion on how the discovered mechanism compares to more complex compositional reasoning tasks (e.g., arithmetic operations where outputs are not present in the input) and to symbolic theories of compositionality.

4. Related work expansion: Please broaden the Related Work section to include:

- studies linking compositionality and modularity in neural networks, and

- circuit discovery via pruning or masking (e.g., Patil et al., NeurIPS 2023; Csordás et al., 2020).

If feasible, provide a short quantitative comparison between the model’s generated outputs and the underlying task distribution, to further support claims about compositional generalization.

Once these issues are addressed, the manuscript should meet PLOS ONE’s publication criteria for technical soundness and transparency.

[Note: HTML markup is below. Please do not edit.]

Reviewer's Responses to Questions

**Comments to the Author**

1. Is the manuscript technically sound, and do the data support the conclusions?

The manuscript must describe a technically sound piece of scientific research with data that supports the conclusions. Experiments must have been conducted rigorously, with appropriate controls, replication, and sample sizes. The conclusions must be drawn appropriately based on the data presented.

Reviewer #1: Partly

Reviewer #2: Yes

**********

2. Has the statistical analysis been performed appropriately and rigorously?

Reviewer #1: No

Reviewer #2: N/A

**********

3. Have the authors made all data underlying the findings in their manuscript fully available?

The PLOS Data policy requires authors to make all data underlying the findings described in their manuscript fully available without restriction, with rare exception (please refer to the Data Availability Statement in the manuscript PDF file). The data should be provided as part of the manuscript or its supporting information, or deposited to a public repository. For example, in addition to summary statistics, the data points behind means, medians and variance measures should be available. If there are restrictions on publicly sharing data—e.g. participant privacy or use of data from a third party—those must be specified.

Reviewer #1: Yes

Reviewer #2: Yes

**********

4. Is the manuscript presented in an intelligible fashion and written in standard English?

PLOS ONE does not copyedit accepted manuscripts, so the language in submitted articles must be clear, correct, and unambiguous. Any typographical or grammatical errors should be corrected at revision, so please note any specific errors here.

Reviewer #1: Yes

Reviewer #2: Yes

**********

5. Review Comments to the Author

Please use the space provided to explain your answers to the questions above. You may also include additional comments for the author, including concerns about dual publication, research ethics, or publication ethics. (Please upload your review as an attachment if it exceeds 20,000 characters)

Reviewer #1: The paper studies representations of compositional structures in transformer-based architectures, with contributions in mechanistic interpretation and in understanding the learned representations of transformer models. The authors employ a sophisticated form of causal ablation, and do multiple experiments employing different attention heads. The generative process which they use for their dataset consists of symbol to colour mappings via a compositional reordering function. The authors randomly generate ten thousand examples for the training set, and two thousand examples for the test set, however they are able to extract a compositional generative process from their model, showing that the task was learned. To perform the extraction, the authors then measure the causal effect of connected nodes via recursive mean-ablating, either by testing one or all-but-one nodes which allows them to identify and extract long chains of circuits even in noisy conditions. Finally, the authors measure R^2 as a means to capture the amount of explained variance.

The authors identify the important nodes for their task and categorize their behaviour into a cohesive algorithm that allows them to reproduce the training data. The main results seem to belong belong to a single trained model, i.e. one instantiation of a transformer with one set of random weights rather than a report of multiple overlapping circuits across runs. The latter case would make a much stronger paper. The additional experiment with different hyperparameters that finds a functionally similar local optimum adds validity, as the authors are able to identify a circuit and extract the same algorithm using their two testing modes. The authors also evaluate their results in the function composition task, although it is not immediately clear if their methodology could capture a circuit for arithmetic reasoning as in [1]. Given the large number of training epochs and datapoints, one could still argue that the transformer only compressed the data into a generative process rather than learned how to compositionally generate data, as the authors also adress with the specialized compositional generalization section. There one could compare data generated by the transformer decoder to the training data in order to evaluate how much of the task distribution was captured.

The paper contributes a new way to identify circuits for the mechanistic interpretation of LLMs, and the discussion could be strengthened by comparing to circuit discovery via pruning (e.g. [2]) or masking (e.g. [3]).

Given the possibility that the paper discusses two models trained on the same task, and that the arising algorithm, although causally validated, is interpreted ad-hoc, the current abstract and intro make too broad claims. However, if the authors match the claims to those of the limitations section, I recommend the paper for publication.

[1] Lake, B. M., & Baroni, M. (2023). Human-like systematic generalization through a meta-learning neural network. Nature, 623(7985), 115-121.

[2]Malakarjun Patil, S., Michael, L., & Dovrolis, C. (2023). Neural Sculpting: Uncovering hierarchically modular task structure in neural networks through pruning and network analysis. Advances in Neural Information Processing Systems, 36, 18495-18531.

[3]Csordás, R., van Steenkiste, S., & Schmidhuber, J. (2020). Are neural nets modular? inspecting functional modularity through differentiable weight masks. arXiv preprint arXiv:2010.02066.

Reviewer #2: Summary:

This paper investigates a specific setting where a compact transformer model displays compositional generalization. The task in this setting is a sequence to sequence arithmetic task (as described by the authors). Through causal analysis of the output predictions, the authors identify specific attention heads contributing to the output. These attention heads, when analyzed, reveal a circuit that systematically retrieves and combines knowledge to solve the task in context. The key finding is that rather than encoding specific functions or operations, the model achieves compositionality through a position-based routing of tokens. The circuit's operation was validated through causal ablation studies and targeted interventions that could predictably alter the model's behavior by manipulating position embeddings.

Strengths:

The methodology is rigorous, combining several previously proposed circuit analysis methods at each step to validate their hypothesis

The paper is very clear. The writing combined with the figures give a clear picture of the circuit learned by the model. Helpful step-by-step visualizations

The focus on compositional generalization and mechanistic interpretability. Two of the most important domains in recent literature

Weaknesses:

Limited scope, which the authors acknowledge

The findings may not be generalizable. Although dynamic routing is a major component in any of the compositionally generalizable solutions, the task set-up is such that it can be solved only with routing. The output answer tokens are already available in the input prompt. A counter example would be learning operations like addition or subtraction where a few output answer tokens may not be available in the inputs. The encoding of the actual operation or function in the model weights may be crucial in such tasks

The related works section remains a bit limited. Expansion is necessary both to compositional generalization literature and findings. Also the intersection of compositional generalization and interpretability (modularity).

Questions:

In the dataset used, do the different episodes (10,000 and 2,000) represent different operations? For example, given S is a specific ordering of the input primitive-color tokens, are the 12,000 episodes unique orderings?

Previous work has shown that compositional generalization usually arises from the diversity in the training dataset. Have the authors experimented with different training dataset sizes? A possible direction to strengthen the work would be to explore training with different number of episodes and checking for circuit consistency

Could the authors elaborate on how this mechanism relates to classical symbolic approaches to compositionality?

**********

6. PLOS authors have the option to publish the peer review history of their article (what does this mean?). If published, this will include your full peer review and any attached files.

If you choose “no”, your identity will remain anonymous but your review may still be made public.

**Do you want your identity to be public for this peer review?** For information about this choice, including consent withdrawal, please see our Privacy Policy.

Reviewer #1: No

Reviewer #2: No

**********

[NOTE: If reviewer comments were submitted as an attachment file, they will be attached to this email and accessible via the submission site. Please log into your account, locate the manuscript record, and check for the action link "View Attachments". If this link does not appear, there are no attachment files.]

To ensure your figures meet our technical requirements, please review our figure guidelines: https://journals.plos.org/plosone/s/figures

You may also use PLOS’s free figure tool, NAAS, to help you prepare publication quality figures: https://journals.plos.org/plosone/s/figures#loc-tools-for-figure-preparation.

NAAS will assess whether your figures meet our technical requirements by comparing each figure against our figure specifications.

# Author comment: Author response to Decision Letter 1

**DOI:** 10.1371/journal.pone.0340088.r002

3 Dec 2025

Please see the corresponding rebuttal file

# Editor report: Decision Letter 1

**DOI:** 10.1371/journal.pone.0340088.r003

**Authors:** Constantine Dovrolis

16 Dec 2025

Circuit Explained: How Does a Transformer Perform Compositional Generalization

PONE-D-25-47812R1

Dear Dr. Jazayeri,

We’re pleased to inform you that your manuscript has been judged scientifically suitable for publication and will be formally accepted for publication once it meets all outstanding technical requirements.

Within one week, you’ll receive an e-mail detailing the required amendments. When these have been addressed, you’ll receive a formal acceptance letter and your manuscript will be scheduled for publication.

An invoice will be generated when your article is formally accepted. Please note, if your institution has a publishing partnership with PLOS and your article meets the relevant criteria, all or part of your publication costs will be covered. Please make sure your user information is up-to-date by logging into Editorial Manager at Editorial Manager® and clicking the ‘Update My Information' link at the top of the page. For questions related to billing, please contact billing support.

If your institution or institutions have a press office, please notify them about your upcoming paper to help maximize its impact. If they’ll be preparing press materials, please inform our press team as soon as possible -- no later than 48 hours after receiving the formal acceptance. Your manuscript will remain under strict press embargo until 2 pm Eastern Time on the date of publication. For more information, please contact onepress@plos.org.

Kind regards,

Constantine Dovrolis

Academic Editor

PLOS One

Additional Editor Comments (optional):

Reviewers' comments:

# Editor report: Acceptance letter

**DOI:** 10.1371/journal.pone.0340088.r004

**Authors:** Constantine Dovrolis

PONE-D-25-47812R1

PLOS One

Dear Dr. Jazayeri,

I'm pleased to inform you that your manuscript has been deemed suitable for publication in PLOS One. Congratulations! Your manuscript is now being handed over to our production team.

At this stage, our production department will prepare your paper for publication. This includes ensuring the following:

* All references, tables, and figures are properly cited

* All relevant supporting information is included in the manuscript submission,

* There are no issues that prevent the paper from being properly typeset

You will receive further instructions from the production team, including instructions on how to review your proof when it is ready. Please keep in mind that we are working through a large volume of accepted articles, so please give us a few days to review your paper and let you know the next and final steps.

Lastly, if your institution or institutions have a press office, please let them know about your upcoming paper now to help maximize its impact. If they'll be preparing press materials, please inform our press team within the next 48 hours. Your manuscript will remain under strict press embargo until 2 pm Eastern Time on the date of publication. For more information, please contact onepress@plos.org.

You will receive an invoice from PLOS for your publication fee after your manuscript has reached the completed accept phase. If you receive an email requesting payment before acceptance or for any other service, this may be a phishing scheme. Learn how to identify phishing emails and protect your accounts at https://explore.plos.org/phishing.

If we can help with anything else, please email us at customercare@plos.org.

Thank you for submitting your work to PLOS ONE and supporting open access.

Kind regards,

PLOS ONE Editorial Office Staff

on behalf of

Dr. Constantine Dovrolis

Academic Editor

PLOS One
